# Positive Association between High Protein Food Intake Frequency and Physical Performance and Higher-Level Functional Capacity in Daily Life

**DOI:** 10.3390/nu14010072

**Published:** 2021-12-24

**Authors:** Mika Kimura, Ai Moriyasu, Hyuma Makizako

**Affiliations:** 1 Center for Health Promotion, International Life Sciences Institute, Tokyo 102-0083, Japan; amoriyasu@ilsijapan.org; 2 Department of Physical Therapy, School of Health Sciences, Faculty of Medicine, Kagoshima University, Kagoshima 890-8544, Japan; makizako@health.nop.kagoshima-u.ac.jp

**Keywords:** protein, physical performance, dietary variety, functional capacity, older adults

## Abstract

Nutritional factors, including low protein intake and poor dietary variety, affect age-associated impairment in physical performance resulting in physical frailty. This cross-sectional study investigated the association between intake frequency of major high protein foods and both physical performance and higher-level functional capacity using the food frequency score (FFS) and high protein food frequency score (PFFS) among community-dwelling older adults. The data of 1185 older adults categorized into quartiles based on FFS and PFFS were analyzed. After adjusting for covariates, FFS and PFFS were significantly associated with physical performance [FFS, usual gait speed (*p* for trend = 0.007); PFFS, usual gait speed (*p* for trend < 0.001), maximum gait speed (*p* for trend = 0.002), timed up and go (*p* for trend = 0.025)], and higher-level functional capacity [FFS (*p* for trend < 0.001); PFFS (*p* for trend < 0.001)]. After excluding PFFS data, the participants’ scores were associated with only higher-level functional capacity. Multi-regression analysis with higher-level functional capacity as the covariate showed that FFS and PFFS were significantly correlated with physical performance. Hence, improving food intake frequency, particularly that of high protein foods, and dietary variety may help maintain higher-level functional capacity and physical performance in community-dwelling older adults.

## 1. Introduction

Reducing the incidence of physical frailty remains an important challenge worldwide. Physical frailty typically increases with age, with frailty among older adults being associated with increased risk of disability, hospitalization, falls, and death [1,2,3,4,5]. According to Fried et al., the criteria for physical frailty, which includes slowness (slow walking speed), weakness (low handgrip strength), low physical activity, exhaustion, and weight loss, involves two physical performance measures—slowness (slow walking speed) and weakness (low handgrip strength) [1]. Several studies have indicated that nutritional factors (status), such as malnutrition, low protein intake, and low vitamin intake, can negatively affect age-associated impairment of physical performance resulting in physical frailty [6,7,8,9,10,11,12]. Malnutrition further increases an individual’s risk of developing sarcopenia and has been associated with low physical performance [8,13].

In particular, dietary protein intake, which is necessary for muscle protein synthesis, has been the focus of several epidemiologic investigations [14,15]. A previous study suggested that greater dietary protein may help slow age-related loss of lean muscle mass [16], and a recent meta-analysis showed a positive dose–response relationship between protein intake and muscle mass increase [17].

These results indicate that good dietary habits are key to reducing age-associated impairment of physical performance and therefore physical frailty. From a public health perspective, the importance of adequate protein intake should be communicated to community-dwelling older adults using simple and easy to understand instructions.

One simple dietary recommendation is dietary variety, with several countries including this in their recommended dietary guidelines, which have been customized to suit unique, local dietary cultures. In Japan, a frequently used recommendation for community-dwelling older adults is the daily intake of 10 food groups, including 5 high protein foods, such as meat, fish/shellfish, eggs, milk/dairy products, and soybeans/soy products, to ensure a balanced diet [18,19,20,21]. Yokoyama et al. have shown that dietary variety based on the intake frequency of the aforementioned 10 food groups was positively associated with physical performance [22,23].

The present study focused on not only dietary variety but also high protein food intake frequency. Given the association between protein intake and physical performance, higher intake frequency of high protein foods might be associated with better physical performance.

To develop easily understandable messaging for community-dwelling older adults, assessing the association between intake frequency and physical performance is important. Additionally, providing data on the association between dietary protein intake and higher-level functional capacity, including instrumental activities of daily living (IADL), would be very useful given that this is one of the indicators of independence and is also associated with mortality and medical and long-term care costs [24,25,26].

The current study therefore sought to investigate the association between the intake frequency of high protein foods and both physical performance and higher-level functional capacity among community-dwelling older adults. 

## 2. Materials and Methods

### 2.1. Study Design and Participants

**Figure 1:**
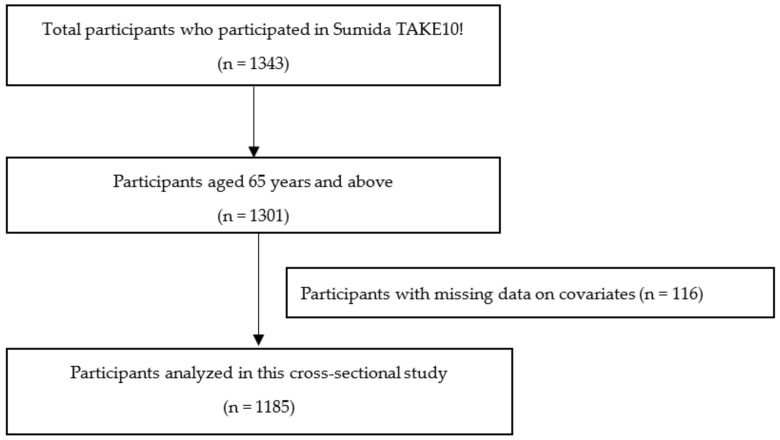
Flow chart of participant inclusion.

This cross-sectional study analyzed data obtained from Sumida TAKE10!, a health intervention session conducted for community-dwelling older adults living in Sumida Ward, Tokyo, Japan. Participants were recruited through ward bulletins and participated in a baseline survey including a questionnaire of food intake frequency and physical performance measurements. This study used data from the baseline survey performed from 2005 to 2018. Among the 1343 participants who participated in Sumida TAKE10!, 1301 participants aged 65 years and above were evaluated for the association between food intake frequency and physical performance. After excluding those with missing data on covariates and food intake frequency, data from 1185 participants remained (Figure 1). Owing to incomplete data on usual gait speed (n = 10), maximum gait speed (n = 13), hand grip strength (n = 4), the timed up and go tests (n = 232), 1175, 1172, 1181, and 954 final data points were obtained for usual gait speed, maximum gait speed, hand grip strength, and timed up and go tests.

This study was approved by the Kagoshima University (Faculty of Medicine) Ethics Committee (Ref No. 190183), and informed consent was obtained from all participants prior to the sessions. Regarding secondary use of data, information was disclosed on our website.

### 2.2. Measures

#### 2.2.1. Physical Performance

Measures of physical performance included 5-m usual walking time, 5-m maximum walking time, hand grip strength (HGS), and timed up and go test (TUG). These measures were reported to be predictive of subsequent mortality, disability, and dependence among older adults [27,28,29,30]. The TUG test is a type of physical performance test in which participants are asked to stand up from a seated position, walk to a cone 3 m away, walk around the cone, walk 3 m back, and return to the sitting position as quickly as possible. The amount of time needed to complete all of these tasks was measured [30]. Usual gait speed and maximum gait speed were each calculated from the 5 m walking time.

#### 2.2.2. Frequency of Food Intake

The intake frequency of 10 food groups, namely meat, fish and shellfish, eggs, milk and dairy products, soybean products, green and yellow vegetables, potatoes, fruits, seaweed, and fats and oils were obtained via a questionnaire. There were four choices for food intake frequency in each food group: (1) eat almost every day (3 points), (2) eat 3 or 4 days a week (2 points), (3) eat 1 or 2 days a week (1 point), and (4) hardly ever eat (0 points). The food frequency score (FFS), which evaluates dietary variety, was calculated as the sum of the scores for each of the 10 food groups (range 0–30) [18]. The high protein food frequency score (PFFS) was calculated as the sum of the scores for meat, fish and shellfish, eggs, milk and dairy products, and soybean products (range 0–15). A score obtained by subtracting the PFFS from FFS was also calculated in order to exclude PFFS data (herein referred to as ex-PFFS, range 0–15). Participants were then categorized into quartiles based on their FFS, PFFS, and ex-PFFS. The FFS was categorized into four groups: Q1 (0–18), Q2 (19–22), Q3 (23–25), and Q4 (26–30). Similarly, the PFFS was categorized into Q1 (0–9), Q2 (10–11), Q3 (12–13), and Q4 (14–15), whereas the ex-PFFS was categorized into Q1 (0–9), Q2 (10–11), Q3 (12), and Q4 (13–15).

#### 2.2.3. Higher-Level Functional Capacity

Higher-level functional capacity was measured using the Tokyo Metropolitan Institute of Gerontology Index of Competence (TMIG-IC), a multidimensional 13-item index comprising three subscales measuring instrumental self-maintenance (IADL, 5 items), intellectual activity (4 items), and social roles (4 items) [23]. A “yes” (able to do) and “no” answer to each item was scored as 1 and 0, respectively. The TMIG Index of Competence has been verified for validity and reliability and is widely accepted and used across Japan as an indicator of independence [23].

#### 2.2.4. Covariates

Height and weight were measured at the baseline survey, from which the body mass index (BMI) was calculated. Demographic characteristics (age and sex), preexisting conditions (hypertension, diabetes, cardiovascular disease, and cerebrovascular disease), and fall incidence within 1 year were determined through interviews with nurses and researchers. The questionnaire included the following five choices to determine weekly exercise habits: (1) do every day, (2) do 5 or 6 days a week, (3) do 2 to 4 days a week, (4) a day a week or less, and (5) none.

### 2.3. Statistical Analysis

The participants’ characteristics were compared according to the quantiles of FFS, PFFS, and ex-PFFS using one-way analyses of variance for continuous variables and the Mantel–Haenszel Test or Kruskal–Wallis Test for categorical variables.

Analysis of covariance was used to assess mean differences in physical performance and higher-level functional capacity (TMIG-IC) according to FFS, PFFS, and ex-PFFS quantiles adjusted for age, sex, survey year, BMI, preexisting conditions, fall incidence within one year, and exercise habits. Multiple comparisons were conducted using the Bonferroni Method. The Jonckheere-Terpstra test was used to determine the significance of a trend (detect and evaluate the trends for continuous values).

To clarify the association between food intake frequency (FFS, PFFS, and ex-PFFS) and physical performance and TMIIG-IC, multiple regression analysis was performed with physical performance (usual gait speed, maximum gait speed, HGS, and TUG) and TMIG-IC as the dependent variables, and food intake frequency (FFS, PFFS, and ex-PFFS) as the explanatory variables. The covariates were age, sex, survey year, BMI, preexisting conditions, fall incidence within 1 year, and exercise habits in Model 1. To clarify whether the effects of food intake frequency on physical performance was mediated by TMIG-IC, TMIG-IC was added as a covariate in Model 2.

All statistical analyses were performed using IBM SPSS Statistics version 27 (IBM Tokyo, Tokyo, Japan). All reported values were two-tailed, with a *p* value of <0.05 indicating statistical significance.

## 3. Results

The demographic characteristics of the participants according to FFS, PFFS, ex-PFFS are summarized in Table 1. Participants had a mean age of 74 years (SD 5.5) and a mean BMI of 23.2 (SD 3.5).

Significant differences in sex, preexisting conditions, falls incidence, and exercise habits were observed among the groups. Notably, participants with higher FFS, PFFS, and ex-PFFS had lower fall incidences; those with higher PFFS had lower preexisting conditions; and those with higher ex-PFFS had more preexisting conditions and better exercise habits.

Table 2 details the adjusted means for physical performance and TMIG-IC according to FFS, PFFS, and ex-PFFS. Notably, PFFS was significantly associated with usual gait speed (*p* for trend < 0.001), maximum gait speed (*p* for trend = 0.002), TUG (*p* for trend = 0.025), TMIG-IC (*p* for trend < 0.001), intellectual activity (*p* for trend < 0.001), and social role (*p* for trend < 0.001). FFS was significantly associated with usual gait speed (*p* for trend = 0.007), TMIG-IC (*p* for trend < 0.001) intellectual activity (*p* for trend < 0.001), and social role (*p* for trend < 0.001). ex-PFFS was significantly associated only with TMIG-IC (*p* for trend < 0.001), intellectual activity (*p* for trend < 0.001), and social role (*p* for trend < 0.001).

Adjusted regression coefficients for physical performance and TMIG-IC per increase in FFS, PFFS, and ex-PFFS are presented in Table 3. FFS and PFFS were significantly correlated with all variables of physical performance and TMIG-IC, whereas ex-PFFS was significantly correlated with three variables of physical performance (without HGS) and TMIG-IC. In the analysis with TMIG-IC as a covariate, no significant correlation was observed between ex-PFFS and all physical performance (Table 4). 

## 4. Discussion

The present study showed that higher PFFS was significantly associated with better physical performance and higher-level functional capacity. In contrast, ex-PFFS was only weakly associated with physical performance but significantly associated with higher-level functional capacity. Although our findings showed that an association between FFS and physical performance was found, the association between PFFS and physical performance was even stronger. These results suggested that high protein food intake frequency was associated with physical performance and higher-level functional capacity, whereas dietary variety was associated with higher-level functional capacity. To the best of our knowledge, this has been the first report to suggest that increasing major high protein food intake frequency may help maintain physical performance and higher-level functional capacity.

Although several mechanisms may contribute to the age-related decrease in skeletal muscle, dietary protein intake is necessary to prevent muscle wasting and maintain skeletal muscle mass and function [31,32,33]. The association between dietary protein intake and lean mass change has been reported in community-dwelling older adults [16]. Moreover, a recent meta-analysis of randomized controlled trials showed a dose–response relationship between protein intake and muscle mass increase [17]. The aforementioned review indicated that protein supplementation was significantly effective in increasing lean muscle mass with or without resistance training. Furthermore, previous studies have suggested an association between physical performance and dietary protein intake [9,10,14]. The present study found that PFFS was associated with physical performance. This indicated that the intake frequency of high protein food was associated with physical performance, which may suggest a strong overall association between dietary protein intake and physical performance. Although other studies have reported an association between vitamins or omega-3 fatty acids and physical performance [8,11,12,34], analyzing their impact using only intake frequency of 10 food groups might be difficult.

Several studies have shown that IADL was associated with nutritional status [35,36,37]. In the current study, however, no significant associations were found between food intake frequency and IADL, although FFS, PFFS, and ex-PFFS were found to be associated both intellectual activity and social role. Given that recruitment for health sessions involved subjects traveling to the sessions by themselves, 96.2% of the subjects were assigned full marks for IADL. Therefore, no significant differences in intake frequency levels could be observed. Intellectual and social ADLs are important factors that help older adults maintain social independence. A previous study also showed an association between dietary variety and intellectual activity [38].

Multi-regression analysis suggested that food intake frequency was more strongly associated with TMIG-IC than with physical performance and that ex-PFFS could possibly be associated with physical performance. However, after adjusting for TMIG-IC, no significant correlation was observed. In contrast, FFS and PFFS were significantly correlated with physical performance after adjustment. This indicated that the association between FFS and PFFS and physical performance was independent of TMIG-IC and that the association between FFS and physical performance was highly dependent on PFFS.

In general, dietary variety has been considered to indicate a well-balanced diet, including a sufficient and balanced supply of nutrients. Indeed, previous studies have reported a relationship between dietary variety and age-related outcomes, such as mortality, cognitive function, and frailty, although the evaluation methods differed [39,40,41,42,43,44]. Several reports indicated an association between frailty and the intake of not only protein but also vitamin D, antioxidant nutrients such as vitamins C and E, and dietary total antioxidant capacity [45,46,47,48,49]. FFS, a simple tool for assessing dietary variety, has been significantly associated with malnutrition and frailty [44,50]. On the other hand, TMIG-IC is a comprehensive index that can be used to evaluate the independence of older adults in social life. In addition, studies have shown a relationship between TMIG-IC and sarcopenia, dental health, quality of life, falls, and skeletal muscle mass [51,52,53,54].

A balanced diet is necessary for healthy aging, a concept supported by the association between FFS and TMIG-IC. Additionally, the lower incidence of falls among participants with higher FFS may also support this concept.

However, the major finding of this study was that PFFS and FFS displayed the same association with physical performance and TMIG-IC. In addition, ex-PFFS showed an association with TMIG-IC but a very weak association with physical performance. Therefore, we considered protein to be the critical component linking FFS with physical performance. The results of the current study suggested that dietary variety is a key to healthy aging, although adequate protein intake may most effectively contribute to better physical performance.

The dietary variety score (DVS) along with the FFS has been used as a popular tool in Japan. Studies have reported an association between DVS and physical function, as well as between body composition and intellectual activity [22,23,55]. Although the FFS contains the same 10 food groups as DVS, their calculation methods differ. DVS counts only everyday food groups (maximum 10 points), whereas FFS assigns 1 point for eating 1 or 2 days a week, 2 points for eating 3 or 4 days, and 3 points for eating almost every day (maximum 30 points). FFS could help provide more detailed information and identify any association between high protein food intake frequency and physical performance in this study.

The present study has several limitations worth noting. First, the FFS did not indicate the quantity of food consumed in each food group. Therefore, it was not clear whether the amount of food consumed affected physical performance and higher-level activity capacity. Thus, the results of this study provide only a general guide for community-dwelling older adults. Second, the subjects were not recruited randomly from the community but were instead recruited from current participants of community health sessions who personally applied in response to a ward bulletin. Therefore, 89% of subjects were female and may already have high health awareness. Third, multivariable analyses did not account for other covariates, such as socioeconomic status, cognitive function, other preexisting illnesses, amount of activity, and chewing ability, which might affect dietary habits and physical performance. Although we did include exercise habits instead of the amount of activity, daily activity is generally a more reliable estimate of overall activity among older adults. Finally, the present study was cross-sectional in nature, which prevents the investigation of causal relationships between high protein food intake frequency or dietary variety and physical performance or high-level functional capacity. However, given that several studies have indicated an association between protein intake and age-related function, the results of this study can be used to provide guidance for community-dwelling older adults. Future studies are needed to confirm whether high protein food intake frequency or dietary variety could contribute toward maintaining physical performance and higher-level functional capacity.

## 5. Conclusions

Based on the FFS and PFFS, the current study found that the intake frequency of major high protein foods was associated with better physical performance and higher-level functional capacity. Additionally, dietary variety was associated with higher-level functional capacity among community-dwelling older adults. These results indicated that improving FFS and PFFS may help maintain higher-level functional capacity and physical performance.

## Figures and Tables

**Table 1:** Demographic characteristics of the participants according to the quartiles of the food frequency score, high protein food frequency score, and excluding the high protein food frequency score.

**FFS**	**Overall**	**Q1 (0–18)**	**Q2 (19–22)**	**Q3 (23–24)**	**Q4 (25–30)**	***p***
**Variable**	**(n = 1185)**	**(n = 300)**	**(n = 353)**	**(n = 306)**	**(n = 226)**	
Women, n (%)	1058 (89.3)	251 (83.7)	318 (90.1)	280 (91.5)	209 (92.5)	0.001
Age (years)	74.0 ± 5.5	73.8 ± 5.4	73.7 ± 5.6	73.9 ± 5.5	74.8 ± 5.3	0.095
Height (cm)	151.9 ± 6.9	152.3 ± 7.3	152.1 ± 6.6	151.6 ± 6.5	151.3 ± 7.3	0.342
Weight (kg)	53.4 ± 9.1	54.0 ± 9.5	53.7 ± 8.9	53.1 ± 8.6	52.7 ± 9.5	0.325
BMI (kg/m^2^)	23.1 ± 3.5	23.3 ± 3.6	23.2 ± 3.4	23.1 ± 3.5	23.0 ± 3.4	0.795
Preexisting conditions	616 (52.0)	169 (55.4)	183 (51.3)	142 (46.0)	128 (56.6)	0.665
Exercise habits	419 (35.4)	87 (29.0)	128 (36.3)	119 (38.9)	85 (37.6)	0.062 *
Fall incidence	218 (18.4)	70 (23.1)	63 (17.7)	50 (16.2)	37 (16.4)	0.033
**PFFS**		**Q1 (0–9)**	**Q2 (10–11)**	**Q3 (12–13)**	**Q4 (14–15)**	***p***
**Variable**		**(n = 333)**	**(n = 335)**	**(n = 353)**	**(n = 164)**	
Women, n (%)		285 (85.6)	301 (89.9)	319 (90.4)	153 (93.3)	0.007
Age (years)		73.8 ± 5.5	73.7 ± 5.5	74.2 ± 5.3	74.5 ± 5.6	0.400
Height (cm)		151.9 ± 7.0	152.0 ± 6.8	151.9 ± 6.9	151.6 ± 7.0	0.940
Weight (kg)		53.9 ± 9.5	53.6 ± 8.3	53.2 ± 9.5	53.0 ± 9.1	0.632
BMI (kg/m^2^)		23.3 ± 3.6	23.2 ± 3.3	23.0 ± 3.6	23.0 ± 3.4	0.608
Preexisting conditions		265 (79.6)	152(45.4)	178 (50.4)	43 (26.2)	<0.001
Exercise habits		101 (30.3)	125 (37.3)	126 (35.7)	67 (40.9)	0.115 *
Fall incidence		78 (23.4)	53 (15.8)	65 (18.4)	22 (13.4)	0.016
**ex-PFFS**		**Q1 (0–9)**	**Q2 (10–11)**	**Q3 (12)**	**Q4 (13–15)**	***p***
**Variable**		**(n = 366)**	**(n = 341)**	**(n = 193)**	**(n = 285)**	
Women, n (%)		311 (85.0)	307 (90.0)	177 (91.7)	263 (92.3)	0.002
Age (years)		73.8 ± 5.4	73.6 ± 5.6	74.1 ± 5.3	74.5 ± 5.4	0.215
Height (cm)		152.3 ± 7.3	151.9 ± 6.3	151.3 ± 6.6	151.6 ± 7.3	0.367
Weight (kg)		53.9 ± 9.1	53.8 ± 9.3	52.5 ± 9.1	53.0 ± 9.0	0.228
BMI (kg/m^2^)		23.2 ± 3.5	23.3 ± 3.5	22.9 ± 3.7	23.0 ± 3.3	0.586
Preexisting conditions		191 (52.2)	162 (47.5)	72 (37.3)	197 (69.1)	0.001
Exercise habits		103 (28.1)	135 (39.6)	70 (36.3)	111 (38.9)	0.009 *
Fall incidence		85 (23.2)	51 (15.0)	39 (20.2)	43 (15.1)	0.031

Values are mean ± SD or the number of cases (%). One-way analysis of variance for continuous variables and Mantel–Haenszel test or Kruskal–Wallis test* for categorical variables. Abbreviations: BMI, body mass index; ex-PFFS, excluding high protein food frequency score; FFS, food frequency score; PFFS, high protein food frequency score.

**Table 2:** Adjusted means for physical performance and higher-level functional capacity according to the quartiles of the food frequency score, high protein food frequency score, and excluding the high protein food frequency score.

**FFS**	**Q1 (0–18)**	**Q2 (19–22)**	**Q3 (23–25)**	**Q4 (26–30)**	p *	***p* for Trend**
**Variable**	**(n = 300)**	**(n = 353)**	**(n = 306)**	**(n =226)**		
Usual gait speed (m/s)	1.37 (0.01) ^b1^	1.39 (0.01)	1.41 (0.01)	1.41 (0.01) ^b1^	0.034	0.007
Maximum gait speed (m/s)	1.79 (0.02)	1.83 (0.01)	1.84 (0.02)	1.84 (0.02)	0.060	0.092
HGS (kg)	22.0 (0.2)	22.4 (0.2)	22.6 (0.2)	22.8 (0.2)	0.109	0.481
TUG(s)	5.73 (0.07)	5.65 (0.07)	5.53 (0.07)	5.50 (0.08)	0.100	0.132
TMIG-IC	11.58 (0.08) ^a1,a2,a3^	12.17 (0.07) ^a1,b1^	12.26 (0.07) ^a2^	12.49 (0.09) ^a3,b1^	<0.001	<0.001
IADL	4.94 (0.02)	4.94 (0.01)	4.97 (0.02)	4.95 (0.02)	0.600	0.195
Intellectual activity	3.49 (0.04) ^a1,a2,a3^	3.73 (0.03) ^a1^	3.76 (0.04) ^a2^	3.81 (0.04) ^a3^	<0.001	<0.001
Social roles	3.17 (0.05) ^a1,a2,a3^	3.49 (0.05) ^a1,b1^	3.53 (0.05) ^a2^	3.72 (0.06) ^a3,b1^	<0.001	<0.001
**PFFS**	**Q1 (0–9)**	**Q2 (10–11)**	**Q3 (12–13)**	**Q4 (14–15)**	p *	***p* for Trend**
**Variable**	**(n = 333)**	**(n = 335)**	**(n = 353)**	**(n = 164)**		
Usual gait speed (m/s)	1.36 (0.01) ^b1,b2^	1.40 (0.01)	1.42 (0.01) ^b1^	1.40 (0.02) ^b2^	0.003	<0.001
Maximum gait speed (m/s)	1.78 (0.02) ^b1,b2,b3^	1.84 (0.01) ^b1^	1.85 (0.01) ^b2^	1.86 (0.02) ^b3^	0.002	0.002
HGS (kg)	21.9 (0.2) ^b1,b2^	22.4 (0.2)	22.7 (0.2) ^b1^	23.0 (0.3) ^b 2^	0.019	0.581
TUG (s)	5.83 (0.07) ^b1,b2^	5.57 (0.07)	5.55 (0.07) ^b1^	5.42 (0.10) ^b2^	0.006	0.025
TMIG-IC	11.69 (0.07) ^a1,a2,a3^	12.19 (0.07) ^a1^	12.26 (0.07) ^a2^	12.42 (0.10) ^a3^	<0.001	<0.001
IADL	4.94 (0.04)	4.96 (0.02)	4.96 (0.01)	4.95 (0.02)	0.753	0.214
Intellectual Activity	3.57 (0.04) ^a1,a2,a3^	3.73 (0.03) ^a1^	3.73 (0.03) ^a2^	3.74 (0.05) ^a3^	0.002	<0.001
Social Roles	3.18 (0.05) ^a1,a2,a3^	3.50 (0.05) ^a1,b1^	3.57 (0.05) ^a2^	3.73 (0.07) ^a3,b1^	<0.001	<0.001
**ex-PFFS**	**Q1 (0–9)**	**Q2 (10–11)**	**Q3 (12)**	**Q4 (13–15)**	p *	***p* for Trend**
**Variable**	**(n = 366)**	**(n = 341)**	**(n = 193)**	**(n = 285)**		
Usual gait speed (m/s)	1.38 (0.01)	1.38 (0.01)	1.40 (0.02)	1.42 (0.01)	0.093	0.245
Maximum gait speed (m/s)	1.81 (0.01)	1.84 (0.02)	1.82 (0.02)	1.85 (0.02)	0.281	0.837
HGS (kg)	22.1 (0.2)	22.6 (0.2)	22.3 (0.3)	22.8 (0.2)	0.106	0.171
TUG (s)	5.66 (0.06)	5.69 (0.07)	5.53 (0.09)	5.53 (0.08)	0.289	0.430
TMIG-IC	11.70 (0.07) ^a1,a2,a3^	12.13 (0.07) ^a1,b1^	12.27 (0.09) ^a2^	12.47 (0.08) ^a3,b1^	<0.001	<0.001
IADL	4.95 (0.01)	4.94 (0.02)	4.97 (0.02)	4.96 (0.02)	0.689	0.331
Intellectual Activity	3.51 (0.03) ^a1,a2,a3^	3.71 (0.03) ^a1^	3.79 (0.05) ^a2^	3.83 (0.04) ^a3^	<0.001	<0.001
Social Roles	3.23 (0.05) ^a1,a2,a3^	3.49 (0.05) ^a1,b1^	3.50 (0.06) ^a2^	3.70 (0.05) ^a3,b1^	<0.001	<0.001

Values are mean (SE). *p* * Analysis of covariance; *p* for trend, Jonckheere–Terpstra test. ^a1^ *p* < 0.01, ^a2^ *p* < 0.01, ^a3^ *p* < 0.01, ^b1^ *p* < 0.05, ^b2^ *p* < 0.05, ^b3^ *p* < 0.05; Bonferroni’s multiple comparison test. Adjusted for age, sex, survey year, BMI, preexisting conditions, fall incidence, and exercise habits. Abbreviations: BMI, body mass index; ex-PFFS, excluding high protein food frequency score; FFS, food frequency score; HGS, hand grip strength; PFFS, high protein food frequency score; IADL, instrumental activities of daily living; TMIG-IC, Tokyo Metropolitan Institute of Gerontology-Index of Competence; TUG, timed up and go test.

**Table 3:** Adjusted regression coefficients for physical performance and higher-level functional capacity per increase in the food frequency score, high protein food frequency score, and excluding the high protein food frequency score.

	Usual Gait Speed (m/s)		Maximum Gait Speed (m/s)		HGS (kg)		TUG (s)		TMIG-IC (Point)		IADL (Point)		Intellectual Activity (Point)		Social Roles (Point)	
Model 1	β (SE)	*p*	β (SE)	*p*	β (SE)	*p*	β (SE)	*p*	β (SE)	*p*	β (SE)	*p*	β (SE)	*p*	β (SE)	*p*
FFS	0.106 (0.001)	<0.001	0.101 (0.002)	<0.001	0.064 (0.240)	0.004	−0.091 (0.008)	0.002	0.272 (0.008)	<0.001	0.036 (0.002)	0.218	0.237 (0.004)	<0.001	0.235 (0.006)	<0.001
PFFS	0.100 (0.002)	<0.001	0.123 (0.003)	<0.001	0.069 (0.045)	0.003	−0.112 (0.015)	<0.001	0.216 (0.016)	<0.001	0.007 (0.003)	0.807	0.159 (0.008)	<0.001	0.213 (0.011)	<0.001
ex-PFFS	0.078 (0.002)	0.003	0.062 (0.003)	0.022	0.048 (0.043)	0.032	−0.050 (0.014)	0.088	0.253 (0.015)	<0.001	0.044 (0.003)	0.132	0.244 (0.007)	<0.001	0.198 (0.010)	<0.001

Adjusted for age, sex, survey year, BMI, preexisting conditions, fall incidence within 1 year, and exercise habits. Abbreviations: BMI, body mass index; ex-PFFS, excluding high protein food frequency score; FFS, food frequency score; HGS, hand grip strength; IADL, instrumental activities of daily living; PFFS, high protein food frequency score; TMIG-IC, Tokyo Metropolitan Institute of Gerontology Index of Competence; TUG, timed up and go test.

**Table 4:** Adjusted regression coefficients for physical performance per increase in the food frequency score, high protein food frequency score, and excluding the high protein food frequency score (including higher-level functional capacity as a covariate).

	Usual Gait Speed (m/s)		Maximum Gait Speed (m/s)		HGS (kg)		TUG (s)	
Model 2	β (SE)	*p*	β (SE)	*p*	β (SE)	*p*	β (SE)	*p*
FFS	0.068 (0.001)	0.013	0.062 (0.001)	0.022	0.039 (0.025)	0.092	−0.066 (0.008)	0.008
PFFS	0.074 (0.002)	0.002	0.106 (0.003)	<0.001	0.049 (0.046)	0.041	−0.101 (0.015)	0.001
ex-PFFS	0.040 (0.002)	0.142	0.036 (0.003)	0.200	0.025 (0.044)	0.275	−0.022 (0.014)	0.465

Adjusted for age, sex, survey year, BMI, preexisting conditions, fall incidence within 1 year, exercise habits and TMIG-IC. Abbreviations: ex-PFFS, excluded high protein food frequency score; FFS, food frequency score; HGS, hand grip strength; PFFS, high protein food frequency score; TMIG-IC, Tokyo Metropolitan Institute of Gerontology Index of Competence; TUG, timed up and go test.

## Data Availability

Data sharing not applicable.

## References

[1] Fried L.P., Tangen C.M., Walston J., Newman A.B., Hirsch C., Gottdiener J., Seeman T., Tracy R., Kop W.J., Burke G. (2001). Frailty in Older adults: Evidence for a phenotype. J. Gerontol. Ser. A Biol. Sci. Med. Sci..

[2] Makizako H., Shimada H., Doi T., Tsutsumimoto K., Suzuki T. (2015). Impact of physical frailty on disability in community-dwelling older adults: A prospective cohort study. BMJ Open.

[3] Abizanda P., Romero L., Sánchez-Jurado P.M., Martínez-Reig M., Gómez-Arnedo L., Alfonso S.A. (2013). Frailty and mortality, disability and mobility loss in a Spanish cohort of older adults: The FRADEA Study. Maturitas.

[4] Ensrud K.E., Ewing S.K., Taylor B.C., Fink H.A., Cawthon P.M., Stone K.L., Hillier T.A., Cauley J.A., Hochberg M.C., Rodondi N. (2008). Comparison of 2 Frailty Indexes for Prediction of Falls, Disability, Fractures, and Death in Older Women. Arch. Intern. Med..

[5] Chittrakul J., Siviroj P., Sungkarat S., Sapbamrer R. (2020). Physical Frailty and Fall Risk in Community-Dwelling Older Adults: A Cross-Sectional Study. J. Aging Res..

[6] Ramsey K.A., Meskers C.G.M., Trappenburg M.C., Verlaan S., Reijnierse E.M., Whittaker A.C., Maier A.B. (2020). Malnutrition is associated with dynamic physical performance. Aging Clin. Exp. Res..

[7] Singh D.K.A., Manaf Z.A., Yusoff N.A.M., Muhammad N.A., Phan M.F., Shahar S. (2014). Correlation between nutritional status and comprehensive physical performance measures among older adults with undernourishment in residential institutions. Clin. Interv. Aging.

[8] Lengelé L., Moehlinger P., Bruyère O., Locquet M., Reginster J.-Y., Beaudart C. (2020). Association between Changes in Nutrient Intake and Changes in Muscle Strength and Physical Performance in the SarcoPhAge Cohort. Nutrients.

[9] McLean R.R., Mangano K.M., Hannan M.T., Kiel D.P., Sahni S. (2016). Dietary Protein Intake Is Protective Against Loss of Grip Strength Among Older Adults in the Framingham Offspring Cohort. J. Gerontol. A Biol. Sci. Med. Sci..

[10] Granic A., Mendonça N., Sayer A.A., Hill T.R., Davies K., Adamson A., Siervo M., Mathers J.C., Jagger C. (2018). Low protein intake, muscle strength and physical performance in the very old: The Newcastle 85+ Study. Clin. Nutr..

[11] Houston D.K., Tooze J.A., Neiberg R.H., Hausman D.B., Johnson M.A., Cauley J.A., Bauer D.C., Cawthon P.M., Shea M.K., Schwartz G.G. (2012). 25-Hydroxyvitamin D Status and Change in Physical Performance and Strength in Older Adults: The Health, Aging, and Body Composition Study. Am. J. Epidemiol..

[12] Houston D.K., Cesari M., Ferrucci L., Cherubini A., Maggio D., Bartali B., Johnson M.A., Schwartz G.G., Kritchevsky S.B. (2007). Association between Vitamin D Status and Physical Performance: The InCHIANTI Study. J. Gerontol. A Biol. Sci. Med. Sci..

[13] Beaudart C., Sanchez-Rodriguez D., Locquet M., Reginster J.-Y., Lengelé L., Bruyère O. (2019). Malnutrition as a Strong Predictor of the Onset of Sarcopenia. Nutrients.

[14] Gregorio L., Brindisi J., Kleppinger A., Sullivan R., Mangano K.M., Bihuniak J.D., Kenny A.M., Kerstetter J.E., Insogna K.L. (2014). Adequate dietary protein is associated with better physical performance among post-menopausal women 60–90 years. J. Nutr. Health Aging.

[15] Bartali B., Frongillo E.A., Stipanuk M.H., Bandinelli S., Salvini S., Palli D., Morais J.A., Volpato S., Guralnik J.M., Ferrucci L. (2012). Protein Intake and Muscle Strength in Older Persons: Does Inflammation Matter?. J. Am. Geriatr. Soc..

[16] Houston D.K., Nicklas B.J., Ding J., Harris T.B., Tylavsky F.A., Newman A.B., Lee J.S., Sahyoun N.R., Visser M., Kritchevsky S.B. (2008). Dietary protein intake is associated with lean mass change in older, community-dwelling adults: The Health, Aging, and Body Composition (Health ABC) Study. Am. J. Clin. Nutr..

[17] Tagawa R., Watanabe D., Ito K., Ueda K., Nakayama K., Sanbongi C., Miyachi M. (2021). Dose–response relationship between protein intake and muscle mass increase: A systematic review and meta-analysis of randomized controlled trials. Nutr. Rev..

[18] Kimura M., Moriyasu A., Kumagai S., Furuna T., Akita S., Kimura S., Suzuki T. (2013). Community-based intervention to improve dietary habits and promote physical activity among older adults: A cluster randomized trial. BMC Geriatr..

[19] Hata T., Seino S., Tomine Y., Yokoyama Y., Nishi M., Narita M., Hida A., Shinkai S., Kitamura A. (2021). The effects of the “Tabepo Check Sheet,” which lists 10 food groups, on the dietary variety of older adults in a metropolitan area. Nihon Koshu Eisei Zasshi.

[20] Okabe Y., Seki A., Miyake Y., Kumagai S. (2018). Effects of the dietary program “SHIKKARITABE CheckSheet 12” to improve dietary variety on higher-level functional capacity among community-dwelling older individuals. Nihon Koshu Eisei Zasshi.

[21] Kimura M., Moriyasu A., Kumagai S., Furuna T. (2016). Evaluation of the comprehensive health program “Sumida TAKE10!” for community-dwelling older adults, which aims to prevent or delay the need for long-term nursing care. Nihon Koshu Eisei Zasshi.

[22] Yokoyama Y., Nishi M., Murayama H., Amano H., Taniguchi Y., Nofuji Y., Narita M., Matsuo E., Seino S., Kawano Y. (2016). Association of dietary variety with body composition and physical function in community-dwelling elderly Japanese. J. Nutr. Health Aging.

[23] Yokoyama Y., Nishi M., Murayama H., Amano H., Taniguchi Y., Nofuji Y., Narita M., Matsuo E., Seino S., Kawano Y. (2017). Dietary variety and decline in lean mass and physical performance in community-dwelling older Japanese: A 4-year follow-up study. J. Nutr. Health Aging.

[24] Koyano W., Shibata H., Nakazato K., Haga H., Suyama Y. (1991). Measurement of competence: Reliability and validity of the TMIG Index of Competence. Arch. Gerontol. Geriatr..

[25] Takata Y., Ansai T., Akifusa S., Soh I., Sonoki K., Takehara T. (2007). High-Level Functional Capacity and 4-Year Mortality in an 80-Year-Old Population. Gerontology.

[26] Taniguchi Y., Kitamura A., Nofuji Y., Ishizaki T., Seino S., Yokoyama Y., Shinozaki T., Murayama H., Mitsutake S., Amano H. (2019). Association of Trajectories of Higher-Level Functional Capacity with Mortality and Medical and Long-Term Care Costs Among Community-Dwelling Older Japanese. J. Gerontol. A Biol. Sci. Med. Sci..

[27] Cooper R., Kuh D., Hardy R., Mortality Review Group (2010). FALCon and HALCyon study teams Objectively measured physical capability levels and mortality: Systematic review and meta-analysis. BMJ.

[28] Shinkai S., Watanabe S., Kumagai S., Fujiwara Y., Amano H., Yoshida H., Ishizaki T., Yukawa H., Suzuki T., Shibata H. (2000). Walking speed as a good predictor for the onset of functional dependence in a Japanese rural community population. Age Ageing.

[29] Bohannon R.W. (2008). Hand-Grip Dynamometry Predicts Future Outcomes in Aging Adults. J. Geriatr. Phys. Ther..

[30] Podsiadlo D., Richardson S. (1991). The Timed “Up & Go”: A Test of Basic Functional Mobility for Frail Elderly Persons. J. Am. Geriatr. Soc..

[31] Koopman R., Van Loon L.J.C. (2009). Aging, exercise, and muscle protein metabolism. J. Appl. Physiol..

[32] Landi F., Calvani R., Tosato M., Martone A.M., Ortolani E., Savera G., D’Angelo E., Sisto A., Marzetti E. (2016). Protein Intake and Muscle Health in Old Age: From Biological Plausibility to Clinical Evidence. Nutrients.

[33] Deer R.R., Volpi E. (2015). Protein intake and muscle function in older adults. Curr. Opin. Clin. Nutr. Metab. Care.

[34] Robinson S.M., Jameson K.A., Batelaan S.F., Martin H.J., Syddall H.E., Dennison E.M., Cooper C., Sayer A.A. (2008). Hertfordshire Cohort Study Group Diet and Its Relationship with Grip Strength in Community-Dwelling Older Men and Women: The Hertfordshire Cohort Study. J. Am. Geriatr. Soc..

[35] Wei K., Nyunt M.-S.-Z., Gao Q., Wee S.-L., Yap K.-B., Ng T.-P. (2018). Association of Frailty and Malnutrition With Long-term Functional and Mortality Outcomes Among Community-Dwelling Older Adults: Results From the Singapore Longitudinal Aging Study 1. JAMA Netw. Open.

[36] Bakhtiari A., Pourali M., Omidvar S. (2020). Nutrition assessment and geriatric associated conditions among community dwelling Iranian elderly people. BMC Geriatr..

[37] Han K., Wang S., Jia W., Cao W., Liu M., Yang S., Wang J., He Y. (2020). Serum albumin and activities of daily living in Chinese centenarians: A cross-sectional study. BMC Geriatr..

[38] Otsuka R., Kato Y., Nishita Y., Tange C., Nakamoto M., Tomida M., Imai T., Ando F., Shimokata H., Suzuki T. (2016). Dietary diversity and 14-year decline in higher-level functional capacity among middle-aged and elderly Japanese. Nutrition.

[39] Kobayashi M., Sasazuki S., Shimazu T., Sawada N., Yamaji T., Iwasaki M., Mizoue T., Tsugane S. (2020). Association of dietary diversity with total mortality and major causes of mortality in the Japanese population: JPHC study. Eur. J. Clin. Nutr..

[40] Tao L., Xie Z., Huang T. (2020). Dietary diversity and all-cause mortality among Chinese adults aged 65 or older: A community-based cohort study. Asia Pac. J. Clin. Nutr.

[41] Otsuka R., Nishita Y., Tange C., Tomida M., Kato Y., Nakamoto M., Imai T., Ando F., Shimokata H. (2017). Dietary diversity decreases the risk of cognitive decline among Japanese older adults. Geriatr. Gerontol. Int..

[42] Yin Z., Fei Z., Qiu C., Brasher M.S., Kraus V.B., Zhao W., Shi X., Zeng Y. (2017). Dietary diversity and cognitive function among elderly people: A population-based study. J. Nutr. Health Aging.

[43] Huang W.-C., Huang Y.-C., Lee M.-S., Chang H.-Y., Doong J.-Y. (2021). Frailty Severity and Cognitive Impairment Associated with Dietary Diversity in Older Adults in Taiwan. Nutrients.

[44] Kiuchi Y., Makizako H., Nakai Y., Tomioka K., Taniguchi Y., Kimura M., Kanouchi H., Takenaka T., Kubozono T., Ohishi M. (2021). The Association between Dietary Variety and Physical Frailty in Community-Dwelling Older Adults. Healthcare (Basel).

[45] Halfon M., Phan O., Teta D. (2015). Vitamin D: A Review on Its Effects on Muscle Strength, the Risk of Fall, and Frailty. BioMed Res. Int..

[46] Marcos-Pérez D., Sánchez-Flores M., Proietti S., Bonassi S., Costa S., Teixeira J.P., Fernández-Tajes J., Pásaro E., Valdiglesias V., Laffon B. (2020). Low Vitamin D Levels and Frailty Status in Older Adults: A Systematic Review and Meta-Analysis. Nutrients.

[47] Balboa-Castillo T., Struijk E.A., Lopez-Garcia E., Banegas J.R., Rodríguez-Artalejo F., Guallar-Castillon P. (2018). Low vitamin intake is associated with risk of frailty in older adults. Age Ageing.

[48] Das A., Cumming R.G., Naganathan V., Blyth F., Ribeiro R.V., Le Couteur D.G., Handelsman D.J., Waite L.M., Simpson S.J., Hirani V. (2020). Prospective Associations Between Dietary Antioxidant Intake and Frailty in Older Australian Men: The Concord Health and Ageing in Men Project. J. Gerontol. A Biol. Sci. Med. Sci..

[49] Kobayashi S., Suga H., Sasaki S., Three-generation Study of Women on Diets and Health Study Group (2017). Diet with a combination of high protein and high total antioxidant capacity is strongly associated with low prevalence of frailty among old Japanese women: A multicenter cross-sectional study. Nutr. J..

[50] Tsuji T., Yamamoto K., Yamasaki K., Hayashi F., Momoki C., Yasui Y., Ohfuji S., Fukushima W., Habu D. (2019). Lower dietary variety is a relevant factor for malnutrition in older Japanese home-care recipients: A cross-sectional study. BMC Geriatr..

[51] Tanimoto Y., Watanabe M., Sun W., Sugiura Y., Tsuda Y., Kimura M., Hayashida I., Kusabiraki T., Kono K. (2012). Association between sarcopenia and higher-level functional capacity in daily living in community-dwelling elderly subjects in Japan. Arch. Gerontol. Geriatr..

[52] Moriya S., Tei K., Yamazaki Y., Hata H., Kitagawa Y., Inoue N., Miura H. (2013). Relationships between higher-level functional capacity and dental health behaviors in community-dwelling older adults. Gerodontology.

[53] Kameyama K., Tsutou A., Fujino H. (2016). The relationship between health-related quality of life and higher-level functional capacity in elderly women with mild cognitive impairment. J. Phys. Ther. Sci..

[54] Wakayama S., Fujita Y., Fujii K., Sasaki T., Yuine H., Hotta K. (2021). Skeletal Muscle Mass and Higher-Level Functional Capacity in Female Community-Dwelling Older Adults. Int. J. Environ. Res. Public Health.

[55] Tomioka K., Okamoto N., Kurumatani N., Hosoi H. (2015). Association of Psychosocial Conditions, Oral Health, and Dietary Variety with Intellectual Activity in Older Community-Dwelling Japanese Adults. PLoS ONE.

